# Thymoquinone Selectively Kills Hypoxic Renal Cancer Cells by Suppressing HIF-1α-Mediated Glycolysis

**DOI:** 10.3390/ijms20051092

**Published:** 2019-03-03

**Authors:** Yoon-Mi Lee, Geon-Hee Kim, Eun-Ji Park, Taek-In Oh, Sujin Lee, Sang-Yeon Kan, Hyeji Kang, Byeong Mo Kim, Ji Hyung Kim, Ji-Hong Lim

**Affiliations:** 1 Department of Food Bioscience, College of Biomedical & Health Science, Konkuk University, Chungju 27478, Korea; yoonmilee@kku.ac.kr; 2 Department of Biomedical Chemistry, College of Biomedical & Health Science, Konkuk University, Chungju 27478, Korea; rlarjsgml4@kku.ac.kr (G.-H.K.); peunji0503@kku.ac.kr (E.-J.P.); dk1050@kku.ac.kr (T.-I.O.); 201341532@kku.ac.kr (S.L.); hsb6477@kku.ac.kr (S.-Y.K.); kkang@kku.ac.kr (H.K.); 3 Diabetes and Bio-Research Center, Konkuk University, Chungju 27478, Chungbuk, Korea; 4 Severance Integrative Research Institute for Cerebral & Cardiovascular Diseases (SIRIC), Yonsei University College of Medicine, Seodaemun-gu, Seoul 03722, Korea; bkim2@yuhs.ac; 5 College of Life Sciences and Biotechnology, Korea University, Seoul 02841, Korea; jay_kim@korea.ac.kr

**Keywords:** thymoquinone, HIF-1α, glycolysis, renal cancer

## Abstract

Several reports have shown that thymoquinone (TQ) effectively attenuates angiogenesis in cancer cells, resulting in suppression of tumor growth. However, it is not yet clear whether TQ reduces hypoxia-inducible factor-1α (HIF-1α) expression in hypoxic cancer cells. Here, we found that TQ was a novel HIF-1α inhibitor through hypoxia response element (HRE)-luciferase assay-based large screening by using 502 natural compounds containing chemical library. TQ reduced HIF-1α protein levels in renal cancer cells; however, it did not affect the HIF-1α protein levels in the presence of proteasome inhibitor, MG132, indicating that the reduction effects of TQ on HIF-1α protein are mediated via the ubiquitination-proteasome dependent pathway. TQ boosted HIF-1α protein degradation, and the mechanism was revealed by inhibiting interaction between HSP90 and HIF-1α. TQ suppressed downstream genes of HIF-1α, indicating negative impact of TQ on HIF-1α transcriptional activities. In addition, TQ altered glucose, lactate, and ATP levels, leading to anaerobic metabolic disturbance. TQ induced apoptosis in hypoxic cancer cells as determined by crystal violet staining and flow cytometry for annexin V-stained cells. Taken together, we suggested that TQ is a potential anticancer agent targeting HIF-1α.

## 1. Introduction

Renal cancer has been highly ranked as a cancer exhibiting serious malignancy and poor prognosis due to its metastatic characteristics and resistance to chemotherapy or radiotherapy in renal cancer patients [1]. Although cytokine-based therapy or vascular endothelial growth factor receptor (VEGFR) inhibitors have been used to treat metastatic renal cancer, the trials did not convincingly suggest a complete cure [1,2]. Thus, more effort is needed for the development of effective therapeutic approaches to treat renal cancer.

Thymoquinone (TQ) is a major bioactive component of *Nigella sativa,* also called as black seed or black cumin, and it exerts health benefits via anti-oxidant, anti-inflammatory, anti-microbial, and anti-diabetic effects [3]. In addition, TQ also exhibited protective functions against several pathophysiological disorders, such as cancer and neurodegenerative diseases [3,4,5,6]. In particular, several reports have demonstrated that TQ can be used as a potential agent against cancer by inhibiting proliferation, angiogenesis, migration, and invasion in various cancer cells [7]. Taking into account the anti-cancer mechanism, TQ inhibits phosphorylated mitogen-activated protein kinase (MAPK), Akt, and mammalian target of rapamycin (mTOR) involved in tumorigenesis signaling pathway. Oncogenic transcription factors, such as nuclear factor-κB (NF-κB) and signal transducer and activator of transcription 3 (STAT-3), were downregulated, while carcinogen-metabolizing enzymes, such as cytochrome p450 1A2 (CYP 1A2) and cytochrome p450 3A4 (CYP 3A4), were upregulated by TQ [8]. However, the effects of TQ on HIF-1α action have not been elucidated.

In cancer, hypoxic condition is a common occurrence due to increased oxygen consumption resulting from rapidly growing cells, thus leading to HIF-1α activation. HIF-1α exacerbates tumor growth upon oxygen and nutrient deprivation through transcriptional reprogramming of angiogenesis, anaerobic glycolysis, and invasiveness [9]. HIF-1α is associated with high incidence of cancer, poor prognosis, and resistance to chemotherapy or radiotherapy in cancer patients. Thus, extensive studies have demonstrated that targeting HIF-1α could be a promising anti-cancer therapeutic strategy [10].

Under normoxic condition, HIF-1α protein is hydroxylated by prolyl hydroxylase (PHD), recognized by E3 ligase complex, mainly containing Von-Hippel Lindau protein (pVHL), and is consequently ubiquitinated and destroyed in the 26S proteasome. Since the hydroxylation does not occur under hypoxic condition, HIF-1α can be stabilized. The stabilized HIF-1α enters into nucleus, and dimerizes with HIF-1β, which is constitutively expressed in the nucleus. The heterodimer recognizes hypoxia response element (HRE) containing downstream genes. Then, genes aggravating the cancer are expressed by transcriptional activators, such as p300 and CREB binding protein (CBP) [11].

HSP90 is known to stabilize HIF-1α protein independent of pVHL-mediated ubiquitination. Geldanamycin, an HSP90 inhibitor, induced HIF-1α degradation, thus leading to a reduction in transcriptional activity of HIF-1α [12]. Other HSP90 inhibitors, such as 17-AAG and 17-DMAG, have also been shown to reduce HIF-1α protein levels and its transcriptional activities [13]. The anticancer activity of HSP90 inhibitors has been studied, and targeting HIF-1α can be one of the possible molecular mechanisms.

Because of oxygen deprivation and poor vascularization, rapidly growing cancer cells fail to undergo oxidative phosphorylation, the most effective way to generate ATP after glycolysis. Thus, increased glycolysis for generating ATP utilized for biosynthesis of biomass, such as amino acids, lipids, and nucleotides is an inevitable metabolic reprogramming which causes rapid growth in cancer cells [14]. HIF-1α plays a pivotal role in this metabolic disturbance by elevating the expression of genes involved in glycolysis and glucose transport [15]. Such metabolic reprogramming confers an opportunity for cancer cells to survive under oxygen and nutrient deprivation condition. Pharmacological inhibitors targeting glycolysis are being widely studied, and several glycolysis inhibitors, such as 2-deoxyglucose and Lonidamine, are being explored in the clinical trial studies [16]. In addition, many studies targeting glycolysis are in the preclinical stage [17].

In the present study, we hypothesized that TQ is a potential anti-cancer drug targeting HIF-1α. We investigated whether TQ is sufficient to suppress HIF-1α activity, and what is its molecular mechanism of action in hypoxic renal cancer cells.

## 2. Results and Discussion

### 2.1. Thymoquinone (TQ) is a Potential HIF-1α Inhibitor

**Figure 1:**
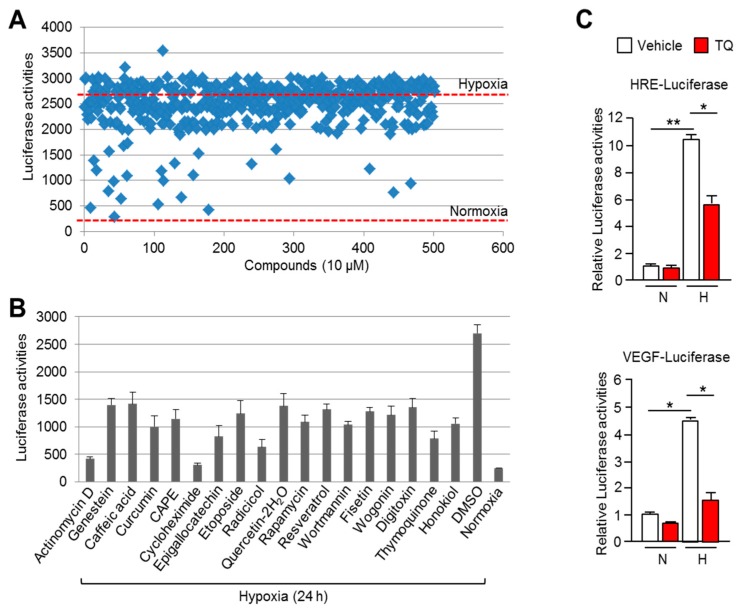
Luciferase assay-based large screening of natural compounds that inhibited Hypoxia-inducible factor-1α (HIF-1α). (**A**) HEK293 cells, transiently expressing HRE-luciferase vector, were incubated with 502 natural compounds dissolved in dimethyl sulfoxide (DMSO). Each compound was used at final concentration of 10 μM for 24 h under hypoxia. (**B**) HEK293 cells expressing HRE-luciferase vector were incubated with 10 μM of 18 compounds for 24 h. The values represent the mean ± SD of three independent experiments performed. (**C**) HRE- and VEGF-luciferase vector was transiently transfected into Caki-1 cells, and then incubated for 24 h to allow stabilization. Cells were further incubated with 10 μM of TQ for 24 h under normoxia or hypoxia. Luciferase activities were normalized using β-gal assay. The values represent the mean ± SD of three independent experiments performed in duplicate; * *p* < 0.05 and ** *p* < 0.01.

Since the development of an HIF-1α inhibitor would be beneficial for cancer therapy, we attempted to identify a potential HIF-1α inhibitor using an HIF-1α-binding sequence containing a hypoxia responsive element (HRE) by conducting a luciferase assay on a library of 502 natural compounds. Here, we identified 18 compounds that suppressed HRE luciferase activity to lower than 50% under hypoxia (Figure 1A). An additional luciferase assay to validate the larger screening also showed that 18 compounds suppressed hypoxia-induced HRE luciferase activities (Figure 1B). Although many of these compounds, including genestein [18], caffeic acid [19], curcumin [20], CAPE [21], epigallocatechin [22], etoposide [23], rapamycin [24], quercetin [25], resveratrol [26], fisetin [27], radicicol [28], wogonin [29], and honokiol [30] have been identified previously as HIF-1α inhibitors, thymoquinone (TQ) was identified as a novel HIF-1α inhibitor (Figure 1B). In addition, Figure 1C shows that TQ suppressed HIF-1α-binding sequence exhibiting VEGF promoter activity (bottom panel) as well as HRE luciferase activity (upper panel) in Caki-1 renal cancer cells under hypoxia. These results revealed that TQ is a potential HIF-1α inhibitor. Although previous reports have shown the anti-angiogenic effect of TQ in cancer, its regulatory mechanism was not clearly elucidated [31]. Here, based on our findings, we can elucidate the unknown regulatory mechanism of TQ-mediated suppression of angiogenesis via HIF-mediated VEGF expression.

### 2.2. TQ Decreases HIF-1α in Hypoxic Renal Cancer Cells

**Figure 2:**
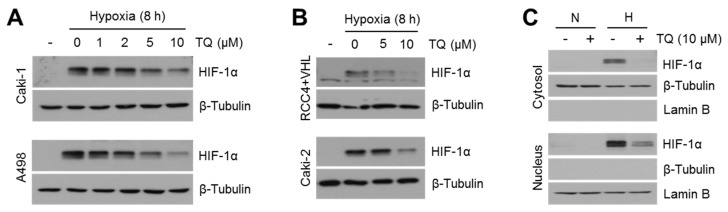
Hypoxia-inducible factor-1α (HIF-1α) is decreased by thymoquinone (TQ) in hypoxic renal cancer cells. (**A**) Caki-1 and A498 renal cancer cells were incubated with various concentrations of TQ (0, 1, 2, 5, or 10 μM) as indicated for 8 h under hypoxia. Protein expression levels were measured by western blotting as described in Materials and Methods. (**B**) RCC4 + pVHL and Caki-2 cells were incubated with TQ as indicated upon hypoxia for 8 h. (**C**) Caki-1 cells treated with 10 μM of TQ upon normoxia or hypoxia for 8 h were used for obtaining cytoplasmic or nuclear protein samples. Nuclear proteins were isolated using high concentration of sodium chloride-based hypertonic buffer. β-tubulin and lamin B proteins were used as a loading control for cytoplasmic proteins and nuclear proteins.

Natural compound screening to identify a potential HIF-1α inhibitor showed that TQ is sufficient to reduce HIF-1α transcriptional activity (Figure 1). In addition, previous reports have shown that TQ suppresses VEGF-mediated angiogenesis during cancer growth [32]. Thus, here we investigated whether hypoxia-inducible factor-1α (HIF-1α) might be a potential target of TQ in renal cancer cells. Initially, we found that TQ efficiently decreases HIF-1α protein levels in a dose-dependent manner in hypoxic Caki-1, Caki-2, A498, and flag-tagged pVHL expressing RCC4 cells (RCC4 + pVHL) (Figure 2A,B). In addition, decreased HIF-1α levels in both cytoplasm and nucleus after treatment with 10 μM of TQ were observed in Caki-1 cells (Figure 2C). These results indicated that TQ decreases HIF-1α protein levels in hypoxic renal cancer cells.

### 2.3. TQ Decreases HIF-1α Expression Independent of PHD-Mediated Hydroxylation but Dependent on Proteasomal Degradation Pathway

**Figure 3:**
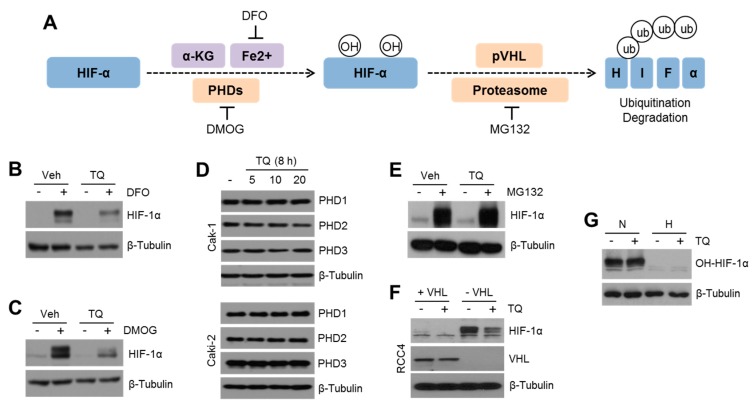
TQ suppresses HIF-1α via the proteasome-dependent degradation pathway. (**A**) Schematic summary of HIF-1α hydroxylation and its proteasome-dependent degradation pathway. Deferoxamine (DFO), as an iron chelator, inhibits HIF-1α hydroxylation. Dimethyloxaloylglycine (DMOG), as a PHDs inhibitor, suppresses HIF-1α hydroxylation. MG132, as a 26S proteasome inhibitor, blocks HIF-1α ubiquitination-mediated degradation. (**B**,**C**) Caki-1 cells were incubated with 10 μM of TQ for 1 h, and then, cells were further incubated for 8 h in the absence or presence of 100 μM of DFO or 0.5 mM of DMOG. (**D**) Caki-1 and Caki-2 cells were cultured with various concentrations of TQ (5, 10, and 20 μM) for 8 h, and then, PHD protein levels were analyzed by western blotting. (**E**) Caki-1 cells were incubated with 10 μM of TQ for 1 h, and then, the cells were further incubated for 6 h in the absence or presence of 20 μM of MG132. (**F**) VHL-null RCC4 (−VHL) and RCC4 (+VHL) cells transiently expressing Flag-tagged VHL were incubated for 8 h with 10 μM of TQ under normoxia. (**G**) Caki-1 cells were incubated with 10 μM of TQ for 1 h, and then, cells were further incubated for 8 h under normoxia or hypoxia. Hydroxylated HIF-1α is measured by using western blotting.

Under hypoxia, HIF-1α is degraded via PHDs-mediated hydroxylation and Von-Hippel Lindau protein (pVHL)-mediated polyubiquitination [11]. Prolyl hydroxylases (PHDs), including PHD1, 2, and 3, enzymatically cause hydroxylation of HIF-1α on proline 402 and 564 residues with co-factors, such as α-ketoglutarate and iron. Dimethyloxaloylglycine (DMOG) and deferoxamine (DFO) are used as PHD inhibitor and iron chelator, respectively. Hydroxylated HIF-1α interacts with pVHL as an E3-ubiquitin ligase, resulting in polyubiquitination-mediated proteasome-dependent degradation, which is blocked by 26S proteasome inhibitor, MG132 (Figure 3A). To understand the molecular mechanism by which TQ suppresses HIF-1α, we initially investigated whether TQ also decreases HIF-1α induced by suppression of hydroxylation. HIF-1α increased by DFO (Figure 3B) or DMOG (Figure 3C) was also significantly decreased in TQ-treated Caki-1 cells, indicating that TQ does not affect PHDs and iron-mediated HIF-1α hydroxylation. In addition, no alteration of PHD1, 2, and 3 was observed in TQ-treated Caki-1 and Caki-2 cells (Figure 3D). Since polyubiquitinated HIF-1α is degraded by 26S proteasome pathway [10], we further investigated whether TQ suppresses MG132-induced HIF-1α. Figure 3E shows that TQ was not sufficient to decrease HIF-1α protein levels accumulated by MG132, demonstrating that TQ may decrease HIF-1α via the proteasome-dependent degradation pathway. An E3-ubiquitin ligase, pVHL, is an important factor involved in polyubiquitination of HIF-1α [10]. Thus, we investigated whether TQ affected pVHL-mediated polyubiquitination of HIF-1α. Here, we found that loss of VHL was sufficient to increase HIF-1α levels under normoxic condition in RCC4 cells, and TQ significantly decreased HIF-1α levels in VHL-null RCC4 cells (Figure 3F). To investigate whether TQ affects HIF-1α hydroxylation under normoxia or hypoxia, hydroxylated HIF-1α (OH-HIF-1α) was measured. Figure 3G shows that hydroxylated HIF-1α was not altered by TQ treatment. These results suggested that TQ suppresses HIF-1α via the proteasome-dependent pathway, but not independent of hydroxylation and pVHL-mediated polyubiquitination pathways.

### 2.4. TQ Suppresses HSP90-Mediated HIF-1α Stabilization

**Figure 4:**
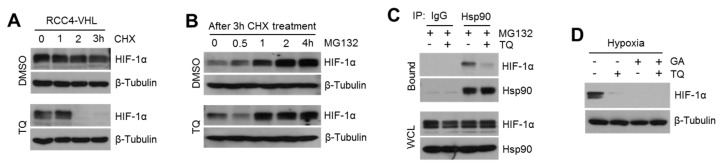
TQ causes rapid degradation of HIF-1α by inhibiting interaction between HIF-1α and HSP90. (**A**) VHL-null RCC4 cells were incubated with 100 μM of CHX for 0, 1, 2, or 3 h in the absence or presence of 10 μM of TQ as indicated. After drug treatment, protein levels were measured by western blotting. (**B**) Caki-1 cells were pre-incubated with 100 μM of CHX for 5 h, and then, blocking of de novo protein synthesis was stopped by replacing of culture medium without CHX. Cells were additionally cultured with 20 μM of MG132 for 0.5, 1, 2, or 4 h in the absence or presence of 10 μM of TQ. (**C**) Caki-1 cells were incubated with 20 μM of MG132 for 6 h in the absence or presence of 10 μM of TQ, and then, total cell lysates were reacted with normal serum or antibodies against for HSP90. After immunoprecipitation, protein complexes were analyzed by western blotting. (**D**) Caki-1 cells were incubated with 10 μM of geldanamycin (GA) for 8 h in the absence or presence of 10 μM of TQ, and then, HIF-1α protein levels were measured by using western blotting.

Figure 3E indicates that TQ decreases HIF-1α levels through the proteasome-dependent degradation pathway. To understand how TQ regulates proteasome-dependent degradation of HIF-1α, we initially evaluated HIF-1α degradation after blocking protein synthesis in the absence or presence of TQ in VHL-null RCC4 cells. Rapid degradation of HIF-1α upon blocking of de novo protein synthesis was observed in TQ-treated VHL-null RCC4 cells, suggesting that reduction in HIF-1α levels in TQ-treated cells is caused by altered degradation pathway (Figure 4A). However, HIF-1α protein accumulation by MG132 was not altered in TQ-treated cells, indicating that de novo synthesis of HIF-1α is not linked to TQ-mediated HIF-1α suppression (Figure 4B). HIF-1α stability is mainly regulated by pVHL-mediated polyubiquitination and HSP90-mediated stabilization [12]. HSP90 inhibitors, such as geldanamycin and radicicol, hindered interaction between HSP90 and HIF-1α, leading to ubiquitin-mediated destabilization of HIF-1α protein [33]. In Figure 4C, we further investigated an interplay between TQ-mediated HIF-1α degradation and HSP90-mediated HIF-1α stabilization. Interestingly, we found that TQ significantly interfered in physical interaction between HIF-1α and HSP90 (Figure 4C). To confirm whether suppression of HIF-1α by TQ is dependent on HSP90-mediated HIF-1α stabilization, TQ effect was measured in the absence or presence of geldanamycin (GA), a selective HSP90 inhibitor. Here, further decreased HIF-1α was not observed under TQ and GA treatment, suggesting that suppression of HIF-1α by TQ may be connected to Hsp90-mediated HIF-1α stabilization (Figure 4D). These results indicated that HSP90-mediated HIF-1α stabilization is a potential target of TQ. Further studies need to unravel the mechanistic action of TQ on interplay between HIF-1α and HSP90.

### 2.5. Expression of Tumor Promoting HIF-1α Target Genes is Downregulated by TQ Treatment

HIF-1α in hypoxic cancer cells upregulated expression of genes linked to tumor progression, such as anaerobic glycolysis (*CA-IX*, *PDK1*, *GLUT1*, and *LDHA*), metastasis (*FN1*, *LOXL2*, and *uPAR*), and angiogenesis (*VEGF*) for cellular adaptation from hypoxic stress [9]. TQ suppressed HIF-1α protein levels, which significantly downregulated the hypoxia-induced tumor promoting HIF-1α target genes, such as *FN1*, *LOXL2*, *uPAR*, *VEGF*, *CA-IX*, *PDK1*, *GLUT1*, and *LDHA*, in TQ-treated Caki-1 (Figure 4A) and Caki-2 (Figure 4B) cells. These results suggested that TQ is sufficient to suppress tumor promoting HIF-1α in hypoxic renal cancer cells.

### 2.6. TQ Suppresses Anaerobic Glycolysis in Hypoxic Renal Cancer Cells

**Figure 5:**
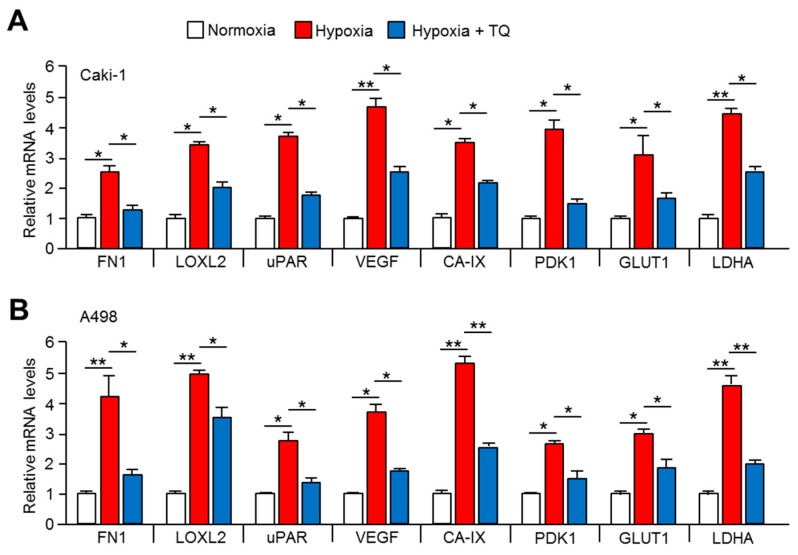
TQ decreases expression of hypoxia-induced HIF-1α target genes in renal cancer cells. (**A**) Caki-1 and (**B**) A498 cells were incubated under normoxia or hypoxia in the absence or presence of 10 μM of TQ for 24 h. The values represent the mean ± SD of three independent experiments performed in duplicate; * *p* < 0.05 and ** *p* < 0.01.

**Figure 6:**
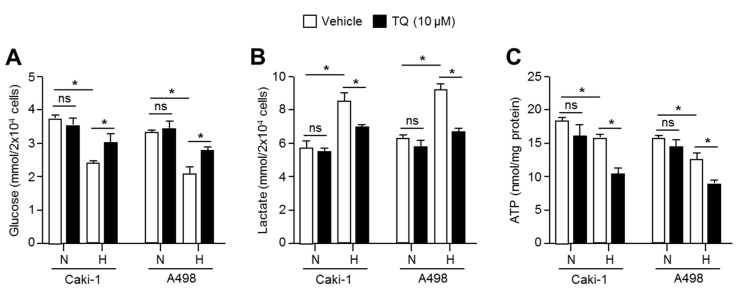
TQ alters glucose-dependent metabolism under hypoxia in renal cancer cells. (**A**) Glucose consumption was measured in Caki-1 and A498 renal cancer cells. Cells were incubated with or without 10 μM of TQ for 24 h under normoxia or hypoxia. (**B**) Lactate production was evaluated. Caki-1 and A498 cells were incubated with or without 10 μM of TQ for 24 h under normoxia or hypoxia. (**C**) Intracellular ATP levels were measured in Caki-1 and A98 cells in the absence or presence of TQ under normoxia or hypoxia. The values represent the mean ± SD of three independent experiments performed in duplicate; * *p* < 0.05.

When cells are exposed to hypoxia, glucose utilization and lactate production is increased through HIF-1α pathway-mediated anaerobic glycolysis to rapidly fulfill biological demands, such as ATP and cellular building blocks, which provides an opportunity for survival and proliferation during metabolic stress caused by hypoxia [13,14,15]. Thus, it is becoming clear that metabolic reprograming of hypoxic cancer cells is a potential target for cancer treatment [16]. Based on the fact that TQ is sufficient to suppress the expression of glycolysis-related genes in hypoxic renal cancer cells (Figure 5), we investigated whether hypoxia-induced metabolic reprogramming to anaerobic glycolysis is targeted in TQ-treated renal cancer cells. Initially, we found that glucose level is decreased in Caki-1 and A498 cultured medium under hypoxia, suggesting that hypoxic renal cancer cells are more addictive to glucose utilization (Figure 6A). Moreover, here we found that TQ significantly increases glucose levels in hypoxic Caki-1 and A498 cultured medium, indicating that hypoxia-induced anaerobic glycolysis is significantly suppressed by TQ treatment (Figure 6A). Since, increased glycolysis causes lactate production in hypoxic cells [13,14,15], we further investigated whether TQ alters hypoxia-induced lactate production and secretion. Consistent with suppression of hypoxic glycolysis by TQ treatment, increased extracellular lactate levels under hypoxia were decreased in TQ-treated Caki-1 and A498 renal cancer cells (Figure 6B). In addition, intracellular ATP levels were significantly decreased in TQ-treated Caki-1 and A498 cells under hypoxia (Figure 6C). These results revealed that hypoxia-induced glucose utilization and lactate production is targeted by TQ in renal cancer cells.

### 2.7. TQ Selectively Promotes Apoptosis in Hypoxic Renal Cancer Cells

**Figure 7:**
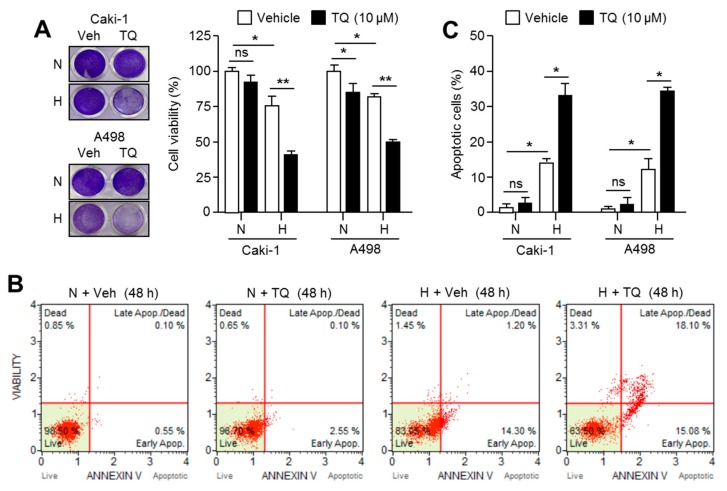
Hypoxic renal cancer cells are more sensitive to TQ-induced apoptosis. (**A**) TQ decreases cell viability in hypoxic renal cancer cells. Caki-1 and A498 cells were incubated with 10 μM of TQ for 48 h under normoxia or hypoxia. Crystal violet staining images are shown (left panel). Quantitative cell viability is also shown (right panel). The values represent the mean ± SD of three independent experiments performed in duplicate; * *p* < 0.05 and ** *p* < 0.01. (**B**,**C**) TQ enhances hypoxia-induced apoptosis in renal cancer cells. Caki-1 and A498 cells were incubated in the absence or presence of 10 μM of TQ for 48 h under normoxia or hypoxia. The values represent the mean ± SD of three independent experiments performed in duplicate; * *p* < 0.05.

HIF-1α mainly triggers cells to evade death caused by metabolic stress upon hypoxia [34]. Since the cancer cells adapted against hypoxic stress exhibit more aggressive behavior, it is necessary to develop pharmacological inhibitors that target hypoxic cancer cells [9]. To provide the clinical application of TQ in cancer treatment, we further investigated whether TQ substantially promotes apoptosis in hypoxic renal cancer cells. Figure 7A shows that TQ significantly decreased cell viability by approximately 50% under hypoxia in Caki-1 and A498 renal cancer cells. Consistently, an increase in the degree of apoptosis by over 15% after TQ treatment under hypoxic condition was observed in Caki-1 and A498 renal cancer cells (Figure 7B and 7C). Tumor hypoxia is closely related to chemotherapy resistance, and HIF-1α induces expressions of multidrug resistance 1 (*MDR1*) and multidrug resistance associated protein 1 (*MRP 1*) genes [35]. Thus, using selective inhibitors targeting hypoxic cancer cells and HIF-1α is an attractive strategy for development of anticancer agents [36], and several reports have shown improved efficacy of anticancer drugs by suppressing HIF-1α under hypoxic condition [37]. Our results additionally revealed that TQ is sufficient to selectively kill hypoxic renal cancer cells. However, further study is needed to validate this action in in vivo.

## 3. Materials and Methods

### 3.1. Reagents and Antibodies

A library of 502 natural compounds was obtained from Enzo Biochem (Farmingdale, NY, USA). Thymoquinone, MG132, geldanamycin (GA), and cycloheximide (CHX) were purchased from Sigma Aldrich (St. Louis, MO, USA). Antibodies recognizing HIF-1α (#3716), Pro564-OH-HIF-1α (#3434), PHD1 (sc-293220), PHD2 (#4835), PHD3 (ab30782), HSP90 (sc-69703), and β-tubulin (sc-9104) were purchased from Cell Signaling Technology (Danvers, MA, USA), Santa Cruz Biotechnology (Dallas, TX, USA), and Abcam (Cambridge, MA, USA).

### 3.2. Cell Culture and Cell Viability Assay

Renal cancer cell lines (Caki-1, Caki-2, A498) were obtained from the Korean Cell Line Bank (Seoul, Korea) and American Type Culture Collection (Manassas, VA, USA), and cultured in McCoy’s 5A and RPMI1640 supplemented with 10% fetal bovine serum. VHL-null RCC4 cell lines were kind gift from Dr. Jong-Wan Park [38], and cultured in Dulbecco’s modified Eagle’s medium (DMEM) supplemented with 10% fetal bovine serum. Cells were incubated in a humidified atmosphere at 37 °C under normoxia (20% O_2_ and 5% CO_2_) or hypoxia (1% O_2_ and 5% CO_2_). Cell viability assay was performed using crystal violet staining [39]. Briefly, cells were cultured in 24-well tissue culture dishes with or without drug treatment. After incubation with drugs, washed cells were fixed using 4% paraformaldehyde, and then stained with 0.5% crystal violet solution for 20 min at room temperature. Optical density of crystal violet was analyzed using 1% SDS solution and measured by an absorbance reader (BioTek, Winooski, VT, USA) (OD570).

### 3.3. Immunoprecipitation and Western Blotting

For immunoprecipitation, cell lysates were prepared using 0.5% CHAPS, 40 mM HEPES (pH 7.5), 150 mM NaCl, and a protease and phosphatase inhibitor cocktail containing CHAPS immunoprecipitation buffer (CIB). One milligram of cell lysates were incubated with 1 μg of antibody recognizing HSP90 and 20 μL of protein G agarose for 16 h at 4 °C. After immunoprecipitation, protein complexes were denatured with 1× sodium dodecyl sulfate (SDS) sample buffer and boiled at 95 °C for 5 min, and then, protein levels were measured using western blotting. For western blotting, total crude proteins were prepared using protein extraction buffer (1% IGEPAL, 150 mM NaCl, 50 mM Tris-HCl (pH 7.9), 10 mM NaF, 0.1 mM EDTA, and a protease inhibitor cocktail). Forty micrograms of proteins were loaded on sodium dodecyl sulfate-polyacrylamide gel electrophoresis (SDS-PAGE), and separated proteins were transferred on PVDF membranes (Millipore, Burlington, MA, USA). Transferred membranes were incubated with primary antibodies (1:1000–1:5000 dilution) against target proteins for 16 h at 4 °C. After primary antibody reactions, membranes were washed using Tween 20 containing Tris-buffered saline (TBS-T), and then, they were incubated with horseradish peroxidase (HRP)-conjugated secondary antibodies (1:10,000) for 1 h at room temperature. For analyzing expression of target proteins, the Enhanced Chemiluminescence (ECL) Prime kit (GE Healthcare, Pittsburgh, PA, USA) was used [39].

### 3.4. Quantitative Real-Time PCR

Quantitative real-time PCR for analyzing mRNA expression was performed using SYBR Green PCR Master Mix (Applied Biosystems, Waltham, MA, USA) as previously described [39]. The primer sequences used in the experiment are shown in Table 1.

### 3.5. Apoptosis Assay

Apoptotic cell numbers were quantitatively analyzed using Muse™ Annexin V and Dead Cell kit (Millipore, Burlington, MA, USA) in accordance with a previously described experimental procedure [39]. Briefly, Caki-1 and A498 cells (1 × 10^5^ cells/well) were seeded and incubated for 24 h to allow stabilization, and then, 10 μM of TQ was treated for 48 h under normoxia or hypoxia. After TQ treatment, collected cells were stained with 100 μL of Muse™ Annexin V and Dead Cell kit reagents (Millipore, Burlington, MA, USA) for 20 min at room temperature. Apoptotic cell population was measured using Mini Flow Cytometry Muse™ Cell Analyzer (Millipore, Burlington, MA, USA).

### 3.6. Luciferase Assay

HIF-1α binding hypoxia-response element (HRE) and VEGF promoter containing luciferase vector (HRE-Luc and VEGF-Luc) were kind gifts from Dr. Jong-Wan Park (Seoul National University) [38]. For luciferase assay, luciferase and β-gal vector were transiently transfected into HEK293 or Caki-1 cells using Lipofectamine 2000 (Invitrogen, Carlsbad, CA, USA) and PolyFect transfection reagent (Qiagen, Venlo, Netherlands). After hypoxia or drug treatment in transfected cells, luciferase activities were measured using a Synergy 2 Luminometer (BioTek, Winooski, VT, USA) and normalized by β-gal assay.

### 3.7. Measurement of Glucose Consumption, ATP, and Lactate Production

Glucose and lactate levels in cultured medium were quantitatively measured using colorimetric assay kit (BioVision, San Francisco, CA, USA) as previously described [40]. Briefly, Caki-1 and A498 cells (1 × 10^5^) were cultured in DMEM without phenol red containing high level of glucose for 24 h in either absence or presence of TQ under normoxia or hypoxia. After incubation, cultured medium was incubated with 50 μL of glucose or lactate assay solution for 30 min at 37 °C upon in dark. Intracellular ATP levels were measured using colorimetric assay kit (BioVision, San Francisco, CA, USA). Briefly, Caki-1 and A498 cells (1 × 10^5^) were cultured in DMEM containing high glucose for 24 h with or without 10 μM of TQ under normoxia or hypoxia, and then, cultured cells were washed and collected into fresh tube. Collected cells were homogenized in 100 μL ATP assay buffer, and then, 20 μL cell lysates in ATP assay buffer were incubated with 50 μL reaction mixture for 30 min at room temperature. After reaction, optical absorbance indicating glucose, lactate, and ATP levels was measured at 570 nm (OD570) using an absorbance reader (BioTek, Winooski, VT, USA).

### 3.8. Statistical Analysis

Data are represented as the mean ± standard deviation (SD), and unpaired Student’s t-test and one-way ANOVA with Tukey posttest were used for statistical analysis. A *p* value of < 0.05 was considered statistically significant.

## 4. Conclusions

The major findings of this study are that 1) thymoquinone (TQ) was identified as an HIF-1α inhibitor using a 502 natural compound library, 2) TQ suppressed hypoxia-induced HIF-1α by suppressing HSP90-mediated stabilization and target genes’ expression, 3) TQ alters hypoxic anaerobic glycolysis and causes metabolic stress, and 4) TQ selectively killed hypoxic renal cancer cells. Overall, our finding suggested that TQ, as an HIF-1α inhibitor, is a potential natural compound involved in clearance of hypoxic renal cancer cells.

## Figures and Tables

**Table 1:** Primer sequences for quantitative real time-PCR.

Gene	Forward Primer	Reverse Primer
FN1	GGTGACACTTATGAGCGTCCTAAA	AACATGTAACCACCAGTCTCATGTG
uPAR	GGTGACGCCTTCAGCATGA	CCCACTGCGGTACTGGACAT
VEGFA	AGCTGCGCTGATAGACATCC	CTACCTCCACCATGCCAAGT
CA-IX	GCGACGCAGCCTTTGAAT	CCACTCCAGCAGGGAAGGA
GLUT1	GGCATTGATGACTCCAGTGTT	ATGGAGCCCAGCAGCAA
PDK1	ATGATGTCATTCCCACAATGGCCC	TGAACATTCTGGCTGGTGACAGGA
LDHA	ACCCAGTTTCCACCATGATT	CCCAAAATGCAAGGAACACT

## Abbreviations

HIF-1α	Hypoxia-inducible factor-1alpha
HRE	Hypoxia response element
VEGFR	Vascular endothelial growth factor receptor
mTOR	Mammalian target of rapamycin
MAPK	Mitogen-activated protein kinase
NF-κB	Nuclear factor-κB
PHD	Prolyl hydroxylase
VHL	Von-hippel linadu
DMOG	Dimethyloxaloylglycine
DFO	Deferoxamine
HSP90	Heat shock protein 90
CA-IX	Carbonic anhydrase 9
PDK1	pyruvate dehydrogenase kinase 1
GLUT1	Glucose transporter 1
VEGF	Vascular endothelial growth factor A
LDHA	Lactate dehydrogenase A
FN1	Fibronectin 1
LOXL2	Lysyl oxidase homolog 2

## References

[1] Shinojima T., Oya M., Takayanagi A., Mizuno R., Shimizu N., Murai M. (2007). Renal cancer cells lacking hypoxia inducible factor (HIF)-1alpha expression maintain vascular endothelial growth factor expression through HIF-2alpha. Carcinogenesis.

[2] Zhang T., Niu X., Liao L., Cho E.A., Yang H. (2013). The contributions of HIF-target genes to tumor growth in RCC. PLoS ONE.

[3] Darakhshan S., Bidmeshki Pour A., Hosseinzadeh Colagar A., Sisakhtnezhad S. (2015). Thymoquinone and its therapeutic potentials. Pharmacol. Res..

[4] Noorbakhsh M.F., Hayati F., Samarghandian S., Shaterzadeh-Yazdi H., Farkhondeh T. (2018). An overview of hepatoprotective effects of thymoquinone. Recent Pat. Food Nutr. Agric..

[5] Farkhondeh T., Samarghandian S., Shahri A.M.P., Samini F. (2018). The neuroprotective effects of thymoquinone: A review. Dose Response.

[6] Samarghandian S., Farkhondeh T., Samini F. (2018). A review on possible therapeutic effect of Nigella sativa and thymoquinone in neurodegenerative diseases. CNS Neurol. Disord. Drug Targets.

[7] Imran M., Rauf A., Khan I.A., Shahbaz M., Qaisrani T.B., Fatmawati S., Abu-Izneid T., Imranh A., Rahman K.U., Gondal T.A. (2018). Thymoquinone: A novel strategy to combat cancer: A review. Biomed. Pharmacother..

[8] Mostofa A.G.M., Hossain M.K., Basak D., Bin Sayeed M.S. (2017). Thymoquinone as a potential adjuvant therapy for cancer treatment: Evidence from preclinical studies. Front. Pharmacol..

[9] Semenza G.L. (2012). Hypoxia-inducible factors: Mediators of cancer progression and targets for cancer therapy. Trends Pharmacol. Sci..

[10] Powis G., Kirkpatrick L. (2004). Hypoxia inducible factor-1alpha as a cancer drug target. Mol. Cancer Ther..

[11] Majkundar A.J., Wong W.J., Simon M.C. (2010). Hypoxia-inducible factors and the response to hypoxic stress. Mol. Cell.

[12] Isaacs J.S., Jung Y.J., Mimnaugh E.G., Martinez A., Cuttitta F., Neckers L.M. (2002). Hsp90 regulates a von Hippel Lindau-independent hypoxia-inducible factor-1 alpha-degradative pathway. J. Biol. Chem..

[13] Ibrahim N.O., Hahn T., Franke C., Stiehl D.P., Wirthner R., Wenger R.H., Katschinski D.M. (2005). Induction of the hypoxia-inducible factor system by low levels of heat shock protein 90 inhibitors. Cancer Res..

[14] Vander Heiden M.G., Cantley L.C., Thompson C.B. (2009). Understanding the Warburg effect: The metabolic requirements of cell proliferation. Science.

[15] Xie H., Simon M.C. (2017). Oxygen availability and metabolic reprogramming in cancer. J. Biol. Chem..

[16] Pelicano H., Martin D.S., Xu R.H., Huang P. (2006). Glycolysis inhibition for anticancer treatment. Oncogene.

[17] Lee Y.M., Lee G., Oh T.I., Kim B.M., Shim D.W., Lee K.H., Jun Y., Lim K.B.O., Lim J.H. (2016). Inhibition of glutamine utilization sensitizes lung cancer cells to apigenin-induced apoptosis resulting from metabolic and oxidative stress. Int. J. Oncol..

[18] Wang G.L., Jiang B.H., Semenza G.L. (1995). Effect of protein kinase and phosphatase inhibitors on expression of hypoxia-inducible factor 1. Biochem. Biophys. Res. Commun..

[19] Jung J.E., Kim H.S., Lee C.S., Park D.H., Kim Y.N., Lee M.J., Park J.W., Kim M.S., Ye S.K., Chung M.H. (2007). Caffeic acid and its synthetic derivative CADPE suppress tumor angiogenesis by blocking STAT3-mediated VEGF expression in human renal carcinoma cells. Carcinogenesis.

[20] Bae M.K., Kim S.H., Jeong J.W., Lee Y.M., Kim H.S., Kim S.R., Yun I., Bae S.K., Kim K.W. (2006). Curcumin inhibits hypoxia-induced angiogenesis via down-regulation of HIF-1. Oncol. Rep..

[21] Choi D., Han J., Lee Y., Chio J., Han S., Hong S., Jeon H., Kim Y.M., Jung Y. (2010). Caffeic acid phenethyl ester is a potent inhibitor of HIF prolyl hydroxylase: Structural analysis and pharmacological implication. J. Nutr. Biochem..

[22] Thomas R., Kim M.H. (2005). Epigallocatechin gallate inhibits HIF-1alpha degradation in prostate cancer cells. Biochem. Biophys. Res. Commun..

[23] Lou J.J., Chua Y.L., Chew E.H., Gao J., Bushell M., Hagen T. (2010). Inhibition of hypoxia-inducible factor-1alpha (HIF-1alpha) protein synthesis by DNA damage inducing agents. PLoS ONE.

[24] Zhong H., Chiles K., Feldser D., Laughner E., Hanrahan C., Georgescu M.M., Simons J.W., Semenza G.L. (2000). Modulation of hypoxia-inducible factor 1ahpha expression by the epidermal growth factor/phosphatidylinositol 3-kinase/PTEN/AKT/FRAP pathway in human prostate cancer cells: Implications for tumor angiogenesis and therapeutics. Cancer Res..

[25] Wilson W.J., Poellinger L. (2002). The dietary flavonoid quercetin modulates HIF-1 alpha activity in endothelial cells. Biochem. Biophys. Res. Commun..

[26] Zhang Q., Tang X., Lu Q.Y., Zhang Z.F., Brown J., Le A.D. (2005). Resveratrol inhibits hypoxia-induced accumulation of hypoxia-inducible factor-1alpha and VEGF expression in human tongue squamous cell carcinoma and hepatoma cells. Mol. Cancer Ther..

[27] Triantafyllou A., Mylonis I., Simos G., Bonanou S., Tsakalof A. (2008). Flavonoids induce HIF-1alpha but impair its nuclear accumulation and activity. Free Radic. Biol. Med..

[28] Hur E., Kim H.H., Choi S.M., Kim J.H., Yim S., Kwon H.J., Choi Y., Kim D.K., Lee M.O., Park H. (2002). Reduction of hypoxia-induced transcription through the repression of hypoxia-inducible factor-1alpha/aryl hydrocarbon receptor nuclear translocator DNA binding by the 90-kDa heat-shock protein inhibitor radicicol. Mol. Pharmacol..

[29] Song X., Yao J., Wang F., Zhou M., Zhou Y., Wang H., Wei L., Zhao L., Li Z., Lu N. (2013). Wogonin inhibits tumor angiogenesis via degradation of HIF-1α protein. Toxicol. Appl. Pharmacol..

[30] Vavilala D.T., Ponnaluri V.K., Vadlapatla R.K., Pal D., Mitra A.K., Mukherji M. (2012). Honokiol inhibits HIF pathway and hypoxia-induced expression of histone lysine demethylases. Biochem. Biophys. Res. Commun..

[31] Peng L., Liu A., Shen Y., Xu H.Z., Yang S.Z., Ying X.Z., Liao W., Liu H.X., Lin Z.Q., Chen Q.Y. (2013). Antitumor and anti-angiogenesis effects of thymoquinone on osteosarcoma through the NF-kB pathway. Oncol. Rep..

[32] Yi T., Cho S.G., Yi Z., Pang X., Rodriguez M., Wang Y., Sethi G., Aggarwal B.B., Liu M. (2008). Thymoquinone inhibits tumor angiogenesis and tumor growth through suppressing AKT and extracellular signal-regulated kinase signaling pathways. Mol. Cancer Ther..

[33] Mabjeesh N.J., Post D.E., Willard M.T., Kaur B., Van Meir E.G., Simons J.W., Zhong H. (2002). Geldanamycin induces degradation of hypoxia-inducible factor 1alpha protein via the proteasome pathway in prostate cancer cells. Cancer Res..

[34] Altman B.J., Rathmell J.C. (2012). Metabolic stress in autophagy and cell death pathways. Cold Spring Harb. Perspect. Biol..

[35] Lv Y., Zhao S., Han J., Zheng L., Yang Z., Zhao L. (2015). Hypoxia-inducible factor-1α induces multidrug resistance protein in colon cancer. Onco Targets Ther..

[36] Sullivan R., Pare G.C., Frederiksen L.J., Semenza G.L., Graham C.H. (2008). Hypoxia-induced resistance to anticancer drugs is associated with decreased senescence and requires hypoxia-inducible factor-1 activity. Mol. Cancer Ther..

[37] Liu P., Wu X., Dai L., Ge Z., Gao C., Zhang H., Wang F., Zhang X., Chen B. (2017). Gambogenic acid exerts antitumor activity in hypoxic multiple myeloma cells by regulation of miR-21. J. Cancer.

[38] Choi Y.J., Shin H.W., Chun Y.S., Leutou A.S., Son B.W., Park J.W. (2016). Diacetoxyscirpenol as a new anticancer agent to target hypoxia-inducible factor 1. Oncotarget.

[39] Kim Y.S., Lee Y.M., Oh T.I., Shin D.H., Kim G.H., Kan S.Y., Kang H., Kim J.H., Kim B.M., Yim W.J. (2018). Emodin sensitizes hepatocellular carcinoma cells to the anti-cancer effect of sorafenib through suppression of cholesterol metabolism. Int. J. Mol. Sci..

[40] Lee G., Won H.S., Lee Y.M., Choi J.W., Oh T.I., Jang J.H., Choi D.K., Lim B.O., Kim Y.J., Park J.W. (2016). Oxidative dimerization of PHD2 is responsible for its inactivation and contributes to metabolic reprogramming via HIF-1α activation. Sci. Rep..

